# Exploitation and Verification of a Stroma- and Metastasis-Associated Risk Prognostic Signature in Pancreatic Adenocarcinoma

**DOI:** 10.3390/ph15111336

**Published:** 2022-10-28

**Authors:** Jia-Hao Zheng, Hong-Fei Yao, Zong-Hao Duan, Pei-Xuan Ji, Jian Yang, Yu-Heng Zhu, Qin-Yuan Jia, Jian-Yu Yang, De-Jun Liu, Yong-Wei Sun, Peng-Cheng Chen, Pei-Dong Shi, Li Chen

**Affiliations:** 1 State Key Laboratory of Oncogenes and Related Genes, Department of Biliary-Pancreatic Surgery, Ren Ji Hospital, School of Medicine, Shanghai Jiao Tong University, Shanghai 200127, China; 2 Shanghai Institute of Digestive Disease, Division of Gastroenterology and Hepatology, Ren Ji Hospital, School of Medicine, Shanghai Jiao Tong University, Shanghai 200001, China; 3 Department of General Surgery, Jiading District Central Hospital Affiliated Shanghai University of Medicine & Health Sciences, Shanghai 201800, China

**Keywords:** pancreatic adenocarcinoma, stroma, metastasis, tumor microenvironment, risk prognostic signature

## Abstract

Pancreatic adenocarcinoma (PAAD), one of the most malignant tumors, not only has abundant mesenchymal components, but is also characterized by an extremely high metastatic risk. The purpose of this study was to construct a model of stroma- and metastasis-associated prognostic signature, aiming to benefit the existing clinical staging system and predict the prognosis of patients. First, stroma-associated genes were screened from the TCGA database with the ESTIMATE algorithm. Subsequently, transcriptomic data from clinical tissues in the RenJi cohort were screened for metastasis-associated genes. Integrating the two sets of genes, we constructed a risk prognostic signature by Cox and LASSO regression analysis. We then obtained a risk score by a quantitative formula and divided all samples into high- and low-risk groups based on the scores. The results demonstrated that patients with high-risk scores have a worse prognosis than those with low-risk scores, both in the TCGA database and in the RenJi cohort. In addition, tumor mutation burden, chemotherapeutic drug sensitivity and immune infiltration analysis also exhibited significant differences between the two groups. In exploring the potential mechanisms of how stromal components affect tumor metastasis, we simulated different matrix stiffness in vitro to explore its effect on EMT key genes in PAAD cells. We found that cancer cells stimulated by high matrix stiffness may trigger EMT and promote PAAD metastasis.

## 1. Introduction

Pancreatic adenocarcinoma (PAAD) is a highly malignant and aggressive tumor which has the 4th highest mortality rate among all cancers [1]. With a 5-year survival rate of only 8%, it poses a serious threat to people’s health [2]. For most PAAD patients, since systemic metastases have often occurred before the time of diagnosis, only a few patients in the early stages can undergo resection, and disease recurrence and metastasis are common and difficult to intervene [3]. Therefore, interventions targeting the metastasis of tumor cells are of significance and there is an urgent need to establish a valid predictive feature to assess the prognosis of PAAD patients to enable patient stratification and precise treatment.

Metastasis is a major cause of high mortality in PAAD patients and is a highly sophisticated process involving angiogenesis/lymphangiogenesis, epithelial mesenchymal transition (EMT), invasion of surrounding tissues, formation of pre-metastatic niches (PMN), and growth of metastatic sites [4]. The interaction between tumor cells and their microenvironment acts as a crucial driver of tumor cell invasion and metastasis [5]. Prior to the onset of tumor cell spread, primary tumors secrete soluble factors and extracellular vesicles to promote PMN formation by providing vascular docking sites for circulating tumor cells, enhancing vascular permeability, remodeling the extracellular matrix, and recruiting immunosuppressive inflammatory cells [6].

Histologically, a distinctive feature of PAAD is the dense fibrotic stroma or desmoplasia found around tumor cells, including overproduction of the extracellular matrix (ECM) and proliferation of stromal cells [7,8]. Desmoplasia leads to poor prognosis by leading PAAD progression and its resistance to chemotherapy [9]. In other words, increased deposition, modification, and remodeling of ECM during tumor progression produces a highly fibrotic tumor microenvironment with increased matrix rigidity [10]. Although the ECM has historically been considered merely a structural scaffold and barrier to tumor cell migration, it is now a growing recognition that stromal stiffness associated with a pro-fibrogenic response can provide biomechanical cues to modulate intracellular signaling pathways and increase tumor malignancy, which is associated with poor patient prognosis, and that some tumor-derived factors may serve as important diagnostic or prognostic biomarkers.

In addition to the complex tumor microenvironment, high heterogeneity and unexplained drug resistance exacerbate the difficulty of pancreatic cancer treatment. Currently, for different clinical stages, gemcitabine-based combination chemotherapy (gemcitabine and capecitabine, albumin paclitaxel, etc.) and modified FOLFIRINOX (fluorouracil, oxaliplatin, irinotecan, leucovorin) therapy represent the standard adjuvant chemotherapy for individuals with resectable and metastatic PDAC. While for borderline resectable PDAC (BRPC) or locally advanced PDAC (LAPC), an individualized treatment plan performed by a multidisciplinary review is recommended [11]. In consideration of the limited efficacy and toxic effects of chemotherapy, targeted drugs and immunotherapeutic agents have recently gained attention and made some progress.

In summary, the link between stromal components and tumor metastasis is critical for PAAD progression. The purpose of this study was to construct a stroma- and metastasis-associated prognostic model for patients with PAAD, so as to accurately predict the prognosis and provide useful recommendations for targeted therapy and immunotherapy.

## 2. Results

**Figure 1:**
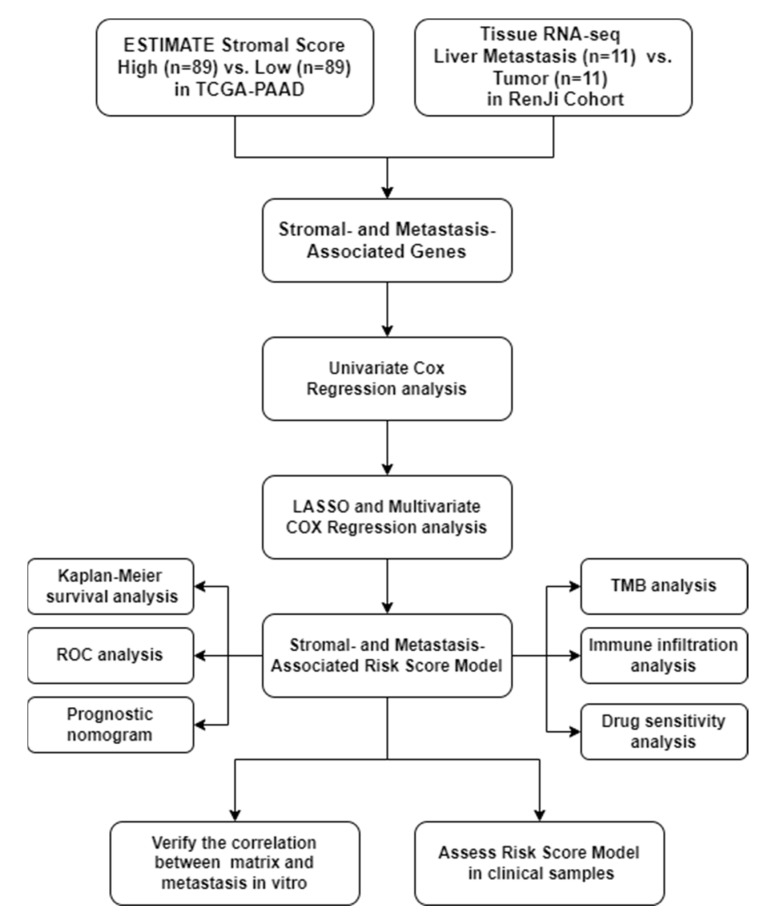
Workflow diagram of this study.

The workflow of this study was as shown in Figure 1.

### 2.1. Exploration of Stroma- and Metastasis-Associated Genes

**Figure 2:**
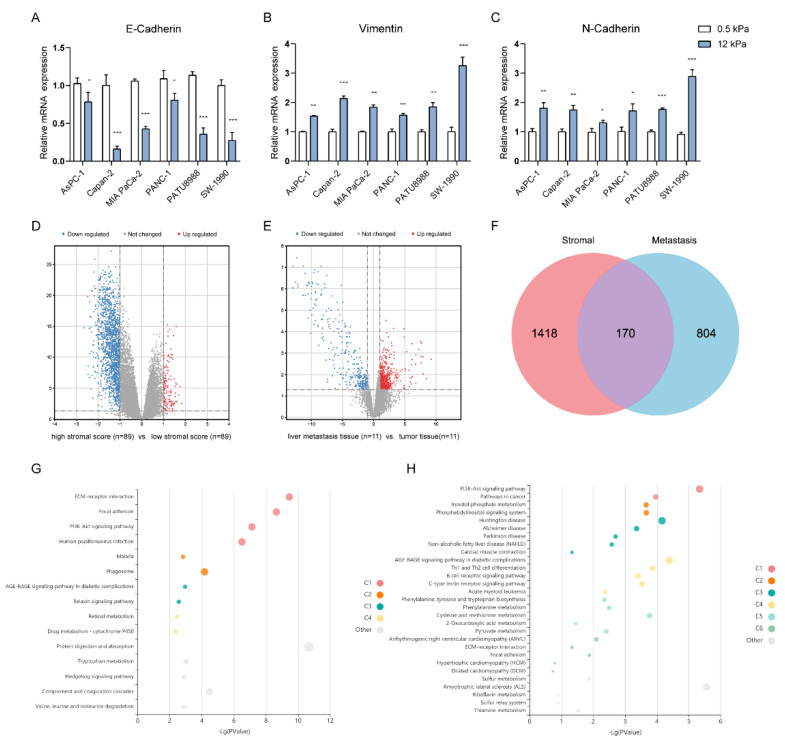
Exploration of Stroma- and Metastasis-Associated Genes. (**A**–**C**) Simulation of the effect of different matrix stiffness on the expression of key genes in EMT In vitro; (**D**) Volcano plot of stroma-associated DEGs based on TCGA-PAAD data; (**E**) Volcano plot of metastasis-associated DEGs based on RenJi cohort transcriptomics data; (**F**) Venn diagrams for screening stroma- and metastasis-associated genes; (**G**) Top 15 GO analysis terms for stroma- and metastasis-associated genes; (**H**) Stroma- and metastasis-associated genes of the top 30 most enriched KEGG pathways. * *p* < 0.05, ** *p* < 0.01, *** *p* < 0.001.

To explore the crosstalk between matrix and metastasis, we used different concentrations of Collagen I Matrigel mixture to simulate the effects of different matrix stiffness (0.5 kPa and 12 kPa) on PAAD cell metastasis. The results showed that the expressions of *N-cadherin* and *Vimentin* were up-regulated in tumor cells stimulated by higher hardness, while the expression of *E-cadherin* showed the contrary (Figure 2A–C). This showed that the related properties of the matrix, especially the stiffness of the matrix, did affect some of the transfer processes.

After that, we intended to screen stroma- and metastasis-associated genes for further study. First, the ESTIMATE algorithm was employed to calculate the stromal score for each PAAD sample in the TCGA database, and the samples were divided into high and low groups based on the score to identify differential genes associated with the stroma component. As shown in the volcano plot about gene expression profiles of the high versus low stromal score groups (Figure 2D), 1588 differentially expressed genes (DEGs) were acquired in the high stromal score group compared with the low score group, named stroma-associated differential genes. Next, we examined the transcriptome microarray data of 11 PAAD liver metastasis cases in the RenJi cohort (liver metastasis tissue vs. tumor tissue) from which we screened for metastasis-related differential genes. As shown in Figure 2E, 974 DEGs were obtained in liver metastases compared with tumor tissues, named metastasis-associated differential genes. Using the Venn algorithm, the two datasets were integrated to obtain a total of 170 DEGs (Figure 2F). The 170 stroma- and metastasis-associated genes were later subjected to gene annotation by KEGG and GO (Figure 2G–H). The GO and KEGG pathway analysis of DEGs showed fascinating results, including the activation of expected matrix-related “ECM-receptor interaction” and “Focal adhesion”, immune-related “Th1 and Th2 cell differentiation” and “B cell receptor signaling pathway” and PI3K/Akt signaling pathways, suggesting that these DEGs affect the prognosis of PAAD by the possible means of activating these pathways, thereby regulating malignant biological behaviors such as proliferation, invasion, and metastasis of tumor cells.

### 2.2. Development of Stroma- and Metastasis-Associated Risk Prognostic Signature

**Figure 3:**
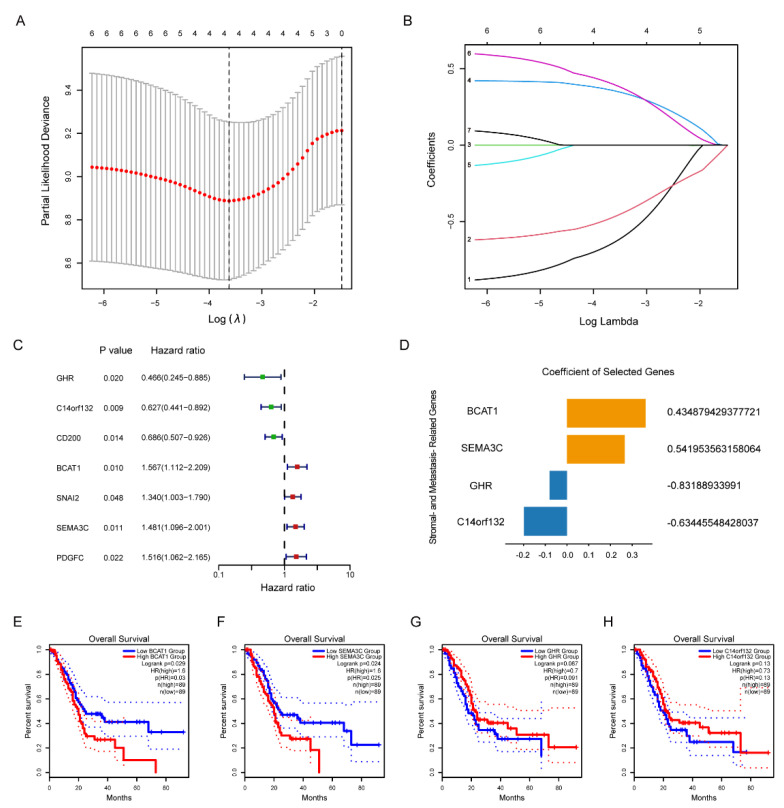
Development of Stroma- and Metastasis-Associated Risk Prognostic Signature. (**A**) Cross-validation plot for penalty term. (**B**) LASSO analysis plot of the stroma- and metastasis-associated genes. (**C**) Forest plot of seven stroma- and metastasis-associated genes. (**D**) Coefficient of the four elected genes. (**E**–**H**) Kaplan–Meier curves of four stroma- and metastasis-associated genes in TCGA database.

At first, we divided the 178 PAAD samples into the training and testing groups. As shown in Supplementary Table S1, there were no differences in clinicopathological characteristics between the two groups. According to the results of univariate Cox regression analysis, LASSO regression analysis was utilized to construct a stroma- and metastasis-associated risk prognostic signature (Figure 3A–C). As a result, seven genes (*GHR*, *C14orf132*, *CD200*, *BCAT1*, *SNAI2*, *SEMA3C*, *PDGFC*) were obtained for further analysis. Subsequently, the multivariate Cox regression analysis was employed to identify the genes having an independent impact on the overall survival of patients, four of which (*GHR*, *BCAT1*, *C14orf132*, *SEMA3C*) were obtained (Figure 3D). Among them, *BCAT1* and *SEMA3C* were poor prognostic factors, and their high expression indicates a short survival time, while the results of *GHR* and *C14orf132* were opposite, indicating that they are protective factors (Figure 3E–H). Finally, through the above operation, an optimal risk prognostic signature based on four genes was developed to predict the outcome of patients. All sample risk scores were calculated according to the following formula: risk score = (−0.8319) × Exp (*GHR*) + (−0.6345) × Exp (*C14orf132*) + 0.4349 × Exp (*BCAT1*) + 0.5420 × Exp (*SEMA3C*). With the counted risk scores, PAAD patients was divided into two groups with discrete OS, namely, high-risk group and low-risk group.

### 2.3. Validation of Stroma- and Metastasis-Associated Risk Prognostic Signature in TCGA

**Figure 4:**
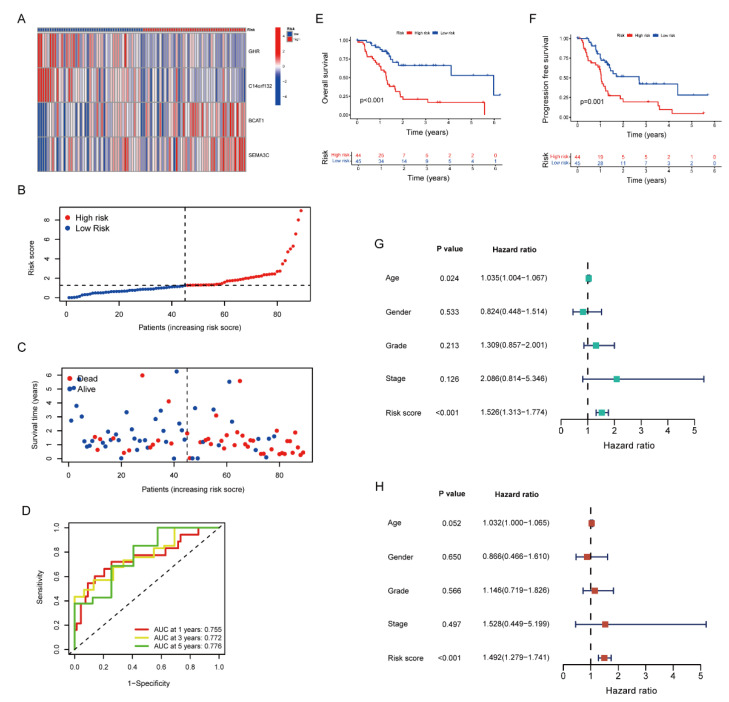
The Prognostic Value of Stroma- and Metastasis-Associated Risk Score Model in the Training Group. (**A**) The heat map illustrated the expression of four genes in each sample. (**B**) The distribution of low- and high-risk samples. (**C**) The correlation of risk score, survival status and survival time (**D**) ROC curve of the risk score. (**E**,**F**) Kaplan–Meier curve of overall survival and progression-free survival in low- and high-risk groups. (**G**,**H**) The univariate and multivariate Cox regression analysis of clinical characteristics and risk score.

Using the scoring formula mentioned above, the risk score of each patient in the training group was counted. The heat map demonstrated the distribution of four genes in each sample (Figure 4A). As the risk score rose, the occurrence of death events gradually increased. (Figure 4B–C). The high-risk group showed a poorer prognosis than that in the low-risk group (*p* < 0.001) (Figure 4E). The progression-free survival rate exhibited the identical tendency (Figure 4F). Moreover, the four genes were utilized in order to establish a highly accurate prognosis scoring system (Figure 4D, AUC_1year_ = 0.755, AUC_2year_ = 0.772, AUC_3year_ = 0.776). The univariate and multivariate Cox regression analysis showed the consistent results, indicating that the increased risk score leads to a worse prognosis (Figure 4G–H).

**Figure 5:**
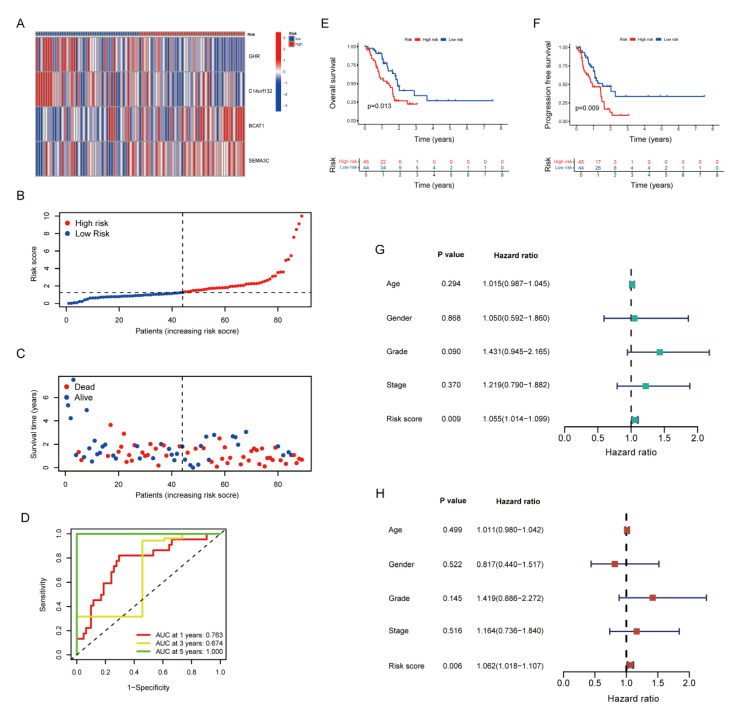
The Prognostic Value of Stroma- and Metastasis-Associated Risk Score Model in the Testing Group. (**A**) The heat map illustrated the expression of four genes in each sample. (**B**) The distribution of low- and high-risk samples. (**C**) The correlation of risk score, survival status and survival time (**D**) ROC curve of the risk score. (**E**,**F**) Kaplan–Meier curve of overall survival and progression-free survival in low- and high-risk groups. (**G**,**H**) The univariate and multivariate Cox regression analysis of clinical characteristics and risk score.

By using an internal testing group and the entire cohort, we verified the stability of the risk signature. Comparing to the training group, the four genes in the testing group and the entire cohort shared similar distributions (Figure 5A and Figure S1A). The higher the risk score, the fewer samples survived and the larger number of dead samples (Figure 5B,C and Figure S1B,C). Additionally, much alike the conclusion of the training group, samples in the high-risk group tended to have worse prognosis in the testing group and the entire cohort (Figure 5E,F and Figure S1E,F). Although differences were found in the ROC curve between the training and testing groups due to the limitation of tumor samples, it still proved that the risk signature had accurate prediction abilities in the testing group and the entire cohort. (Figure 5D and Figure S1D). Indicated by the Cox regression analysis, the risk signature can serve as an independent prognostic factor, showing its potential to predict patient prognosis (Figure 5G,H and Figure S1G,H).

### 2.4. Identifying the Predictive Capability of Risk Signatures for Prognosis

**Figure 6:**
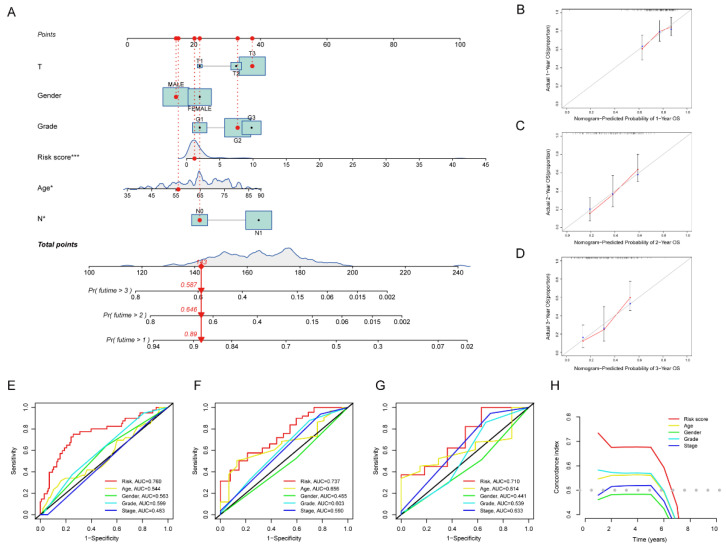
Identifying the Predictive Capability of Risk Signatures for Prognosis. (**A**) Establishment of a nomogram based on the signature to predict the 1-, 2-, and 3-year OS. (**B**) One-year nomogram calibration curves of the TCGA cohort. (**C**) Two-year nomogram calibration curves of the TCGA cohort. (**D**) Three-year nomogram calibration curves of the TCGA cohort. (**E**–**G**) The time-dependent ROC of the nomogram based on the OS. (**H**) The concordance index (c-index) for the risk signature and other clinical characteristics. * *p* < 0.05, *** *p* < 0.001.

First, a nomogram combining the clinical data (sex and stage) of patients and the risk score was conducted to predict the 1-, 2-, and 3-year overall survival of PAAD patients (Figure 6A). After the risk scores were calculated based on the tumor stage and risk score, it was verified that the nomograph was of great prognostic value using calibrate curves (Figure 6B–D). The AUCs of the nomogram were 0.760, 0.737, 0.710 in 1-year, 2-year and 3-year OS (Figure 6E–G). In addition, the results of the concordance index (c-index) also showed that the prediction effect of risk signature was better than others (Figure 6H).

### 2.5. Correlation of Risk Prognosis Signature with Tumor Mutational Burden

**Figure 7:**
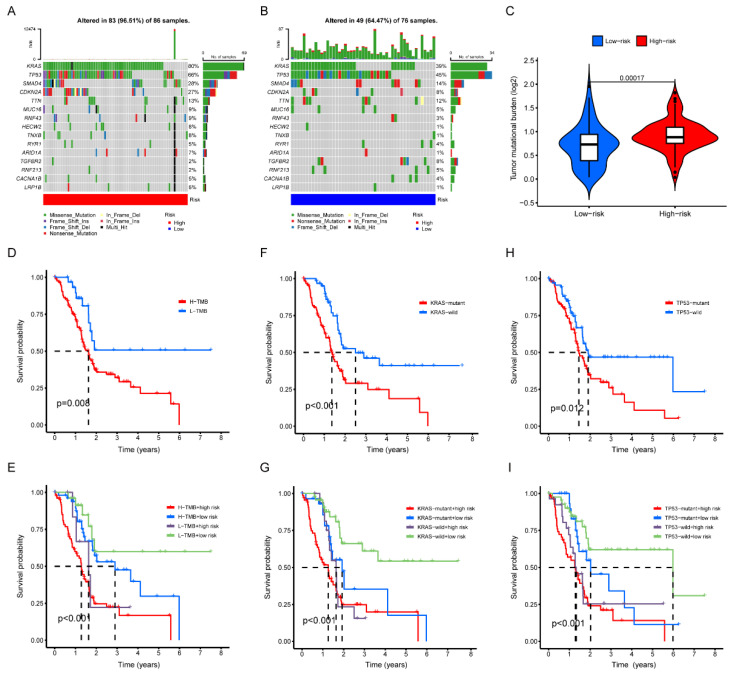
Correlation of Risk Prognosis Signature with Tumor Mutational Burden. (**A**,**B**) The waterfall plots of mutant genes in high- and low-risk groups. (**C**) The Violin plot of TMB score in high- and low-risk groups. (**D**) Kaplan–Meier survival curve of high-TMB and low-TMB. (**E**) Kaplan–Meier survival curve stratified by risk score and TMB. (**F**) Kaplan–Meier survival curve of *KRAS*-mutant and *KRAS*-wild. (**G**) Kaplan–Meier survival curve stratified by risk score and *KRAS*. (**H**) Kaplan–Meier survival curve of *TP53*-mutant and *TP53*-wild. (**I**) Kaplan–Meier survival curve stratified by risk score and *TP53*.

It has been reported that tumor mutational burden (TMB) may be highly correlated with patient survival and has been utilized as a biomarker in certain cancer types to identify patients who would benefit from immunotherapy [12]. In order to verify the difference of TMB between the two groups, the “maftool” package was utilized to analyze and conclude the mutational data acquired from the TCGA. The top 15 mutation genes in the two groups were shown in the waterfall plots (Figure 7A,B), as they were identical in two groups, while the mutation frequencies were different. Moreover, it could be concluded that the *KRAS* and *TP53* were the genes with the top mutation ratios between the two groups. Furthermore, TMB in the high-risk group was higher than that in the low-risk group (*p* < 0.001) (Figure 7C). According to the survival analysis, the survival time of samples was negatively correlated with the high-TMB (*p* = 0.008) (Figure 7D), the *KRAS*-mutant (*p* = 0.001) (Figure 7F) and the *TP53*-mutant (*p* = 0.012) (Figure 7H), respectively. After combining the results of TMB analysis and the risk score, the outcome suggested that the high-TMB and the high-risk tended to have the worst prognosis among the cohort (*p* < 0.001) (Figure 7E). To take this further, we performed a stratified analysis based on risk score, *KRAS* and *TP53* mutation status to prove our theory about whether there could be a collective impact between *KRAS* and *TP53* mutation and the risk score. The results showed that the lower the risk scores and the *KRAS* or *TP53* mutation proportion, the better mortality rate there might be (Figure 7G,I).

### 2.6. Correlation of Risk Prognostic Signature and Tumor Immune Microenvironment

**Figure 8:**
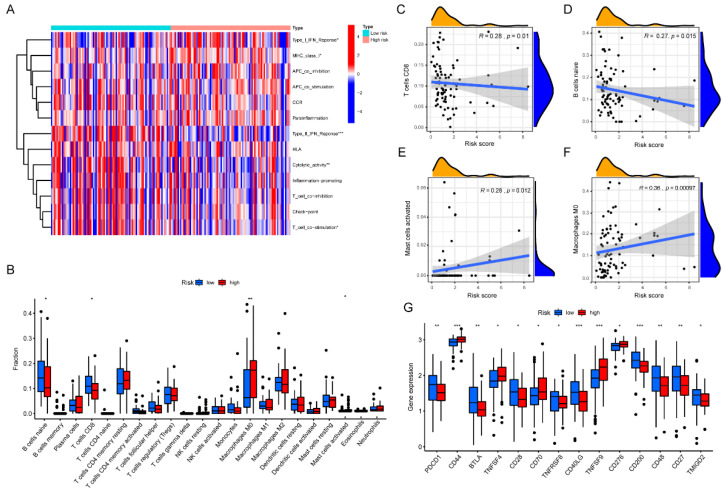
Correlation of Risk Prognostic Signature and Tumor Immune Microenvironment. (**A**) Differences in immune function between the high- and low-risk groups; (**B**) Differences in the proportions of different immune cells in the high- and low-risk groups; (**C**–**F**) Correlation between risk scores and key immune cells; (**G**) Differences in the expression of key molecules of immune checkpoints between the high- and low-risk groups. * *p* < 0.05, ** *p* < 0.01, *** *p* < 0.001.

Numerous studies have shown that cancer-associated stromal cells such as CAFs not only promote tumor proliferation and metastasis, but also induce immune evasion of cancer cells and immunosuppressive effects [13,14] via interactions with the tumor immune microenvironment (TIME) components, especially immune cells [15,16]. Meanwhile, TIME has been reported to be closely related to the clinical prognosis of tumor patients [17], for which we carried out further studies to explore the effect of risk signature in TIME. First, to quantify the enrichment scores of immune-related functions, ssGSEA was adopted to explore the correlation between immune functions and risk signature. The results showed that type I and type II IFN responses, cytolytic activity and T-cell co-stimulation differed prominently between the two groups (Figure 8A). In addition, we used the CIBERSORT algorithm to compare the proportions of different immune cells in the two groups (Figure 8B). It was found that the risk signature was closely related to some subtypes of immune cells, with an increase in the proportion of unpolarized macrophages as well as activated mast cells with increasing risk scores, while the opposite was observed for CD8+T cells and naive B cells (Figure 8C–F). These results suggest that the immune response may be more active in samples with low-risk scores. Overall, the prognostic risk signature was closely related to the immune infiltration of PAAD, and these results may provide new insights into the PAAD tumor microenvironment.

TIME also determines the status of the immune response in TME, which depends mainly on the composition and activity of the infiltrating immune cells, as well as alterations in immune checkpoint molecules [18]. As research about immune checkpoint molecules is being conducted, immune checkpoint inhibitors (ICIs) are increasingly being considered as a method for clinical treatment [19]. We intended to investigate whether there is a correspondence between immune checkpoints and risk signature. Therefore, we selected key molecules including PDCD1 (PD-1), CD44, BTLA, TNFSF4, CD28, CD70, TNFRSF8, CD40LG, TNFSF9, CD276, CD200, CD48, CD27, TMIGD2 to assess their correlations with risk scores (Figure 8G). The results showed that CD44, TNFSF4, CD70, TNFSF9, and CD276 were upregulated in the high-risk group, while the other molecules were reversed. Thus, risk signature may be an influential factor in predicting the outcome of ICIs treatment in PAAD patients.

### 2.7. Correlation of Risk Prognostic Signature and Chemotherapy Drug Sensitivity

**Figure 9:**
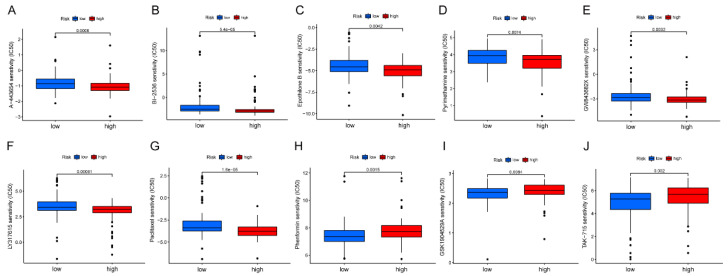
Correlation of Risk Prognostic Signature and Chemotherapy Drug Sensitivity. (**A**–**J**) Differences in sensitivity (IC50) for 10 common chemical drugs between high- and low-risk groups.

In recent decades, with the continuous development of new anti-cancer drugs, tumor chemotherapy has been widely used. However, the current anti-cancer drugs do not possess high sensitivity, and they have damaging effects on normal cells while playing their roles as therapeutic methods. Such problems have gradually become major obstacles limiting the dosage and hindering the efficacy. Therefore, the early prediction of patients’ sensitivity to drugs is essential to ensure successful completion of treatment and improve long-term quality of life [20]. To investigate whether different drugs have different sensitivities in the two groups of high and low risk, we screened hundreds of chemical drugs using the pRRophetic algorithm and selected the top 10 most statistically significant drug candidates (Figure 9A–J). Patients with higher risk scores were sensitive to a smaller proportion of the drugs (Phenformin, GSK1904529A, TAK-715), while those with low-risk scores showed a higher sensitivity to the rest of the drugs (A-443654, BI-2536, Epothilone B, Pyrimethamine, Paclitaxel, GW843682X, LY317615). These findings may provide guidance for clinical drug application in the future.

### 2.8. Verification of the Stroma- and Metastasis-Associated Risk Prognostic Model in Public Database and RenJi Samples

**Figure 10:**
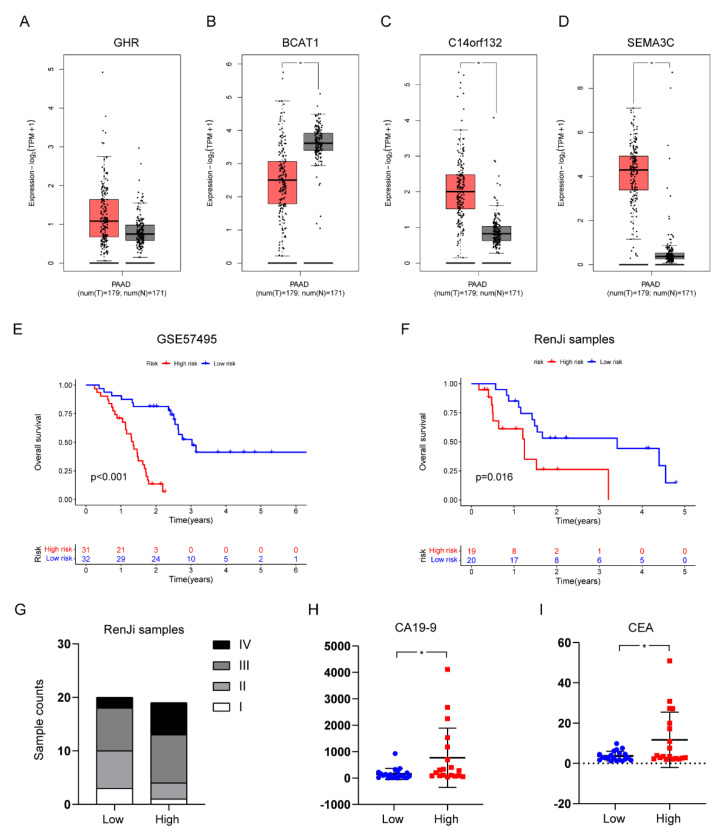
Verification of the Stroma- and Metastasis-Associated Risk Prognostic Model in Public Database and RenJi Samples. (**A**–**D**) The expression profile of the risk gene signature in TCGA and GTEx datasets (tumor tissue was indicated in red and normal tissue in gray); (**E**,**F**) Survival curves regarding high- and low-risk groups in GSE57495 database and RenJi sample; (**G**) Different TNM stage counts in the high- and low-risk groups in RenJi sample; (**H**,**I**) Differences in tumor markers (CA19-9 and CEA) between high- and low-risk groups. * *p* < 0.05.

To identify the expression profile of the risk gene signature, TCGA and Genotype-Tissue Expression (GTEx) datasets were collected and analyzed (Figure 10A–D). We then investigated the prognostic value of the risk signature using the GEO database (GSE57495) (Figure 10E). To further determine the predictive value of the risk prognostic model, we performed real-time PCR on 39 tumor tissues samples from RenJi patients and took the obtained expression values of each gene into the formula described previously. Subsequently, 39 samples were divided into high- and low-risk group according to the risk score. We studied the differences in clinical data such as TNM staging (Figure 10G), CA19-9 (Figure 10H) and CEA (Figure 10I) between the high- and low-risk group. In addition, Kaplan–Meier curves were also used to visually show the difference in survival time between high- and low-risk groups (Figure 10F). Consistent with the expected results, in our tumor samples, the prognosis of the high-risk group was worse than that of the low-risk group (*p* = 0.016).

## 3. Discussion

It is well known that the treatment of PAAD is a daunting challenge. To date, little progress has been made in the early diagnosis and effective treatment of patients with PAAD. Therefore, it is necessary to develop a new prognostic feature from the clinical and biological characteristics of PAAD, which can not only accurately predict the total survival time of patients, but also contribute to the improvement in clinical decision making. In addition, mathematical models in pancreatic cancer research suggest that PAAD does not always progress in a linear fashion but may be the result of a simultaneous increase in genetically altered cells [21]. Therefore, considering the development of prognostic strategies, targeting a single factor may not be sufficient to classify patients with PAAD. As one of the most malignant tumors, PAAD not only has a high mesenchymal component, but is also characterized by an extremely high metastatic risk. This study explored the collaborative effect of stromal and metastatic microenvironments on the prognosis of patients with PAAD.

Studies have shown that the components of the microenvironment in PAAD are associated with poor patient prognosis [7]. The tumor microenvironment of PAAD contains a large amount of dense stromal components, and the aberrant proliferation of stromal cells and abnormal ECM dynamics promote the formation of a tumorigenic microenvironment, leading to malignant transformation, further providing favorable conditions for metastasis [22,23]. Nielson et al. demonstrated that stromal regulation within PAAD liver metastases is distinct and dependent on the interaction of immune components. This process may occur prior to cancer cell metastasis [24]. In terms of exploring the potential mechanism of matrix components affecting tumor metastasis, considering pancreatic cancer as one of the tumors with high matrix stiffness, we preliminarily used the Matrigel mixture with different concentrations of Collagen I to simulate different matrix stiffness. Since EMT often occurs at the initial stage of tumor invasion and metastasis, we explored the expression differences of EMT key genes in different culture environments of 0.5 kPa and 12 kPa. As expected, the expression of E-cadherin significantly decreased in tumor cells stimulated by high stiffness, while the expression of N-cadherin and Vimentin increased. We hypothesize that pancreatic cancer cells may sense high matrix stiffness, which induces EMT and further promotes the formation of the metastatic microenvironment.

In our study, we identified stroma- and metastasis-associated genes using TCGA data and transcriptomic data from RenJi tissue samples, respectively. Integrating the two sets of DEGs, we constructed a risk prognostic model based on four genes (*GHR*, *BCAT1*, *C14orf132*, *SEMA3C*) by Cox regression analysis and LASSO regression analysis. We have retrieved some promising results from the gene signatures above, some of which have been shown to be bonafide candidates involved in tumor pathogenesis. Research by Pedersen et al. found out applying a threshold for positivity to the methylated BCAT1/IKZF1 blood assay could improve the specificity for colorectal cancer recurrence [25]. Moreover, Basu et al. reported that in melanoma cells in vitro, GHR antagonist could downregulate the ATP-binding cassette-containing transporter and consequently sensitize them to anti-cancer drug treatment [26]. In PAAD, research by Zhang et al. proved a close relationship between SEMA3C expression and highly-expressed *KRAS^G12D^* mutation, indicating a possible and attractive target for PAAD patients [27]. Moreover, Xu et al. revealed that SEMA3C overexpression was associated with poor prognosis in PAAD patients through the activation of the ERK1/2 signaling pathway [28]. As expected, patients with high-risk scores had shorter overall survival times than those with low-risk scores, both in the TCGA cohort, the GSE57495 dataset, and the RenJi samples. In addition, this risk profile independently predicted the prognosis of PAAD patients with the optimal predictive power compared to other clinicopathological characteristics. We then detected the expression of several risk genes in the TCGA and GETx databases. There was no significant difference in the expression of GHR between tumors and normal tissues, but studies had shown that GHR was highly expressed in pancreatic cancer and facilitated tumor progression [29,30]. This may be related to confounding factors, such as ethnic and geographical differences. However, it does not prevent us from regarding several risk genes as a holistic model for risk stratification of patients to effectively predict prognosis.

Moreover, we used the ssGSEA and CIBERSORT algorithm to assess the immune-related functions and the proportion of several subtypes of immune cells. In PAAD, the dense content of the extracellular matrix remains to be one of the major physical obstacles rejecting the delivery of anti-tumor drugs, and subsequently constructing a chemotherapy-resistant and immunosuppressive tumor microenvironment [31]. Our results found that in samples with higher risk scores, immune function “MHC class I” were up-regulated and “T cell co-stimulation” were down-regulated, and there were fewer CD8+T cells in the immune cell population, which may indicate that as the risk factor increases, CD8+T cells fail to activate and are in an incompetent state, or even undergo apoptosis to effectively fight tumor formation and progression due to the persistent driving effect of long-term antigen presentation and the lack of costimulatory signals provided by T-cell co-stimulation molecules. This potential cause of CD8+ T cell dysfunction was also mentioned in the review by Philip M et al. [32]. Mast cells have been recognized as central regulators of tissue remodeling and as anterior immune cells that coordinate innate and adaptive immune responses [33]. Moreover, higher mast cell tumor infiltration was associated with a decrease in IFN-γ-producing CD8+ T cells [34], predicting an undesirable response to anticancer therapy [35]. And M0 macrophages were susceptible to TME and rapidly became tumor-promoting M2 phenotypes, which may lead to failure of antitumor treatment and worsening tumor progression [36]. These studies may also support the notion that the increased proportion of M0 macrophages and mast cells in the high-risk group predicts a worse prognosis. Undeniably, a deeper understanding of the multidimensional interactions between tumor-associated stromal cells and infiltrating immune cells within TME will help us better identify the potential molecular targets for stromal cell-targeted therapies.

In the correlation analysis between risk score and drug sensitivity, multiple drugs with different sensitivities in the high- and low-risk groups were identified, such as Paclitaxel, Phenformin, Pyrimethamine, Epothilone B, BI-2536, GW843682X, etc. Epothilone B, a microtubule stabilizing agent, had been reported to block cancer cell division by interfering with microtubule proteins such as paclitaxel [37], and they both showed higher sensitivity in low-risk groups. Polo-like kinase 1 (PLK1) was closely related to cell cycle regulation and could accelerate the proliferation and progression of tumor cells. Moreover, overexpression of PLK1 was associated with poor prognosis of many cancers, making PLK1 an attractive target for cancer treatment [38]. Both BI-2536 and GW843682X were PLK1 inhibitors [39,40], and both were highly sensitive in the low-risk group. Studies had demonstrated that pyrimethamine could inhibit oncogenic proteins such as STAT3 and NF-κB and induce apoptosis of tumor cells when synergized with temozolomide [41]. Among the more sensitive drugs in the high-risk group, phenformin had been shown to act as an authentic tumor disruptor, not only to maintain energy metabolism homeostasis by activating AMP-activated protein kinase (AMPK), but also as a blocker of mTOR regulatory complexes [42]. In other words, choosing different drugs for patients with different risk scores may improve their prognosis.

The limitations of our study are as follows. First, the stroma- and metastasis-related microenvironments vary due to the different sites of tumors. The analysis of tumors as a whole part may lack specificity. Second, the number of PAAD cases in the TCGA and RenJi cohorts used for screening and validation of risk-prognosis models was insufficient. Third, the mechanism of stromal components related to tumor metastasis was not fully explored. Finally, the data for the raw letter analysis were retrospectively obtained from the available data and were prone to selection bias. The above deficiencies will be considered in the further validation of our findings in subsequent studies from the perspectives of multi-omics (single-cell RNA sequencing integrated spatial transcriptomics), multicenter large samples, and prospectively designed experiments, respectively. With the amendments mentioned above, we attempt to explore in depth the still unclear mechanistic studies between stromal components and tumor metastasis.

## 4. Materials and Methods

### 4.1. Data Collection

Gene expression data and clinical survival information were downloaded from the TCGA database (TCGA-PAAD), GTEx database and GEO database (GSE57495). The status of the clinical data of PAAD patients is shown in Table 1, including age, gender, stage, grading and TMN. Samples with no clinical data were excluded from further analysis. In addition, clinical information of the RenJi cohort and RenJi samples is presented in Supplementary Materials Tables S3 and S4.

### 4.2. Establishment and Verification of Risk Prognostic Model

The TCGA-PAAD samples were randomly divided into two groups at a ratio of 1:1, named the training group and the testing group. To define the prognostic correlation genes in the training group, a univariate Cox regression analysis was used for the 170 co-expressed genes. Then, a Least absolute shrinkage and selection operator (LASSO) regression analysis was used to avoid overfitting and remove those closely related genes, and the risk signature were further established based on the results of two-step multivariate Cox regression analysis. The formula for the calculation of the risk score was as follows:

Risk score = Ʃ [Exp (gene) × Coef (gene)]

Exp(gene) denotes the gene expression, while Coef (gene) generation denotes the regression coefficient. In accordance with the risk score, we separated the samples into high-risk and low-risk groups based on the median risk score. The survival analysis, receiver operating characteristic (ROC) curves, and the areas under the time-dependent ROC curves (AUC) were presented using the “survmine” and “survival” packages. Validation of risk prognostic signature was implemented during the testing group and the whole cohort.

### 4.3. Analysis of Independent Prognostic Factors

We performed univariate and multivariate Cox regression analyses in the high- and low-risk groups in order to confirm the applicability of the risk signature and other clinical characteristics as the independent prognostic factors.

### 4.4. Differential Expression Analysis and Its Related Functional Analysis

The DEGs were screened out by the criterion of |log2FoldChange| > 1 and adjusted *p* < 0.05 to explore possible pathways relevant to the risk signature. Subsequently, we applied GO analysis and KEGG pathway analysis to spot the various biological functions and pathways between the two groups, during which the “limma” package was used to identify DEGs, and the enrichment analysis was carried out using the “clusterProfiler”, “enrichplot” and “org.Hs.eg.db” [43].

### 4.5. Tumor Mutational Burden

The corresponding TMB of each sample was obtained from TCGA, and underwent analysis and summarization by the “maftools” package [44]. The results were visualized by waterfall plots. To analyze the survival of different TMB, the “survminer” and “survival” packages were used.

### 4.6. Nomogram and Calibration Curves

The nomogram laying out the risk score and other clinical pathological features was built to establish a viable method for prognostic and overall survival in 1,2- and 3-years prediction of PAAD patients. The calibration curve was utilized to assess and demonstrate the accurateness between predicted results and actual survivals.

### 4.7. Differential Analysis of Tumor Immune Microenvironment

To distinguish the differences between the tumor immune microenvironment of two groups, several analyses were performed. Firstly, we applied the “ESTIMATE” algorithm to evaluate the infiltrating cells and the tumor purity in the tumor microenvironment [45]. Secondly, we employed the “GSEAbase” package to run the ssGSEA, which served as a reflection of the enrichment of several immune functions-related gene sets in two groups [46]. Additionally, the “CIBERSORT” algorithm (https://cibersort.stanford.edu/ (accessed on 14 June 2022)) and the “MCPcounter” package were employed to assess the enrichment of 22 immune cells inside each sample [47,48]. Finally, to evaluate the distinctions between two groups, we retrieved immune checkpoint key molecules from previous literature. Pearson correlation analysis was utilized to check the relevance between risk score and infiltrating immune cells or immune checkpoint key molecules.

### 4.8. Sensitivity Analysis of Chemotherapeutic Agents

Based on the Cancer Genome Project (CGP) gene expression matrix and drug treatment information, the R package of pRRophetic was performed to predict the half maximal inhibitory concentration (IC50) of common chemotherapeutic agents in the TCGA cohort. IC50, as a measure of a drug’s resistance or sensitivity, can reflect the degree of drug tolerance of a certain type of cells. Wilcoxon signed-rank test was used to detect differences between groups [49].

### 4.9. Cell Culture of Human PAAD Cells

Human PAAD cell lines AsPC-1, Capan-2, MIA PaCa-2, PANC-1, Patu8988, and SW-1990 were obtained from the State Key Laboratory of Oncogenes and Related Genes, Ren Ji Hospital, School of Medicine, Shanghai Jiao Tong University. AsPC-1 cells were cultured in Roswell Park Memorial Institute (RPMI) 1640 culture medium, while others were cultured in Dulbecco’s modified Eagle’s medium (DMEM), supplemented with 10% fetal bovine serum (FBS) and 1% streptomycin/penicillin (P/S) at 37 °C in a humidified incubator under 5% CO_2_ condition.

### 4.10. Quantitative Real-Time PCR

For isolating RNA from tumor samples and tumor cells, we used TRIzol (Takara Bio, Dalian, China). Then RNA was transcribed to cDNA using the Prime Script RT Master Mix reagent (Takara Bio, Dalian, China). The primer sequences used in qRT−PCR were demonstrated in the Supplementary Table S2. *18s* was chosen to be an internal reference gene.

### 4.11. RNA Sequencing

Total RNAs were isolated from freshly frozen tumors with RNeasy MinElute Cleanup Kit (Qiagen, Hilden, Germany), and the qualified RNAs were amplified by PCR to construct a cDNA library. After filtering the raw data, the cDNA library was sequenced on the Illumina Hiseq X-Ten platform (Illumina, San Diego, CA, USA). After the raw sequence data were evaluated and quality trimmed, the STAR (v2.5.2b) algorithm was used to map the resulting pure reads to human genome reference (hg19) [50]. Subsequently, according to the previous study, the raw data were further genetically annotated and quantified [51].

### 4.12. Statistical Analysis

In our study, the statistical was performed with R (version 4.0.2; R), and *p* value <0.05 was regarded as significant. We applied student’s *t*-test in this study to compare the differences between the two groups. Kaplan–Meier survival curves were presented to show survival, and Log-rank tests were applied to compare survival differences. The Mann–Whitney U test was utilized to compare the ssGSEA scores. The IC50 of drugs was compared by the Wilcoxon signed-rank test.

## 5. Conclusions

Collectively, our study developed a risk prognostic signature of PAAD by LASSO and Cox regression analysis starting from stroma- and metastasis-related DEGs, and the reliability and validity of this model were verified in multiple datasets and clinical specimens. Critically, the signature not only serves as an important predictor of the prognosis of PAAD patients, but also correlates significantly with the tumor immune microenvironment. In addition, the signature also has a certain auxiliary effect on clinical medication guidance. In conclusion, this study provides a novel and promising way to facilitate individualized survival prediction and develop personalized cancer treatment.

## Figures and Tables

**Table 1:** Clinical pathological parameters of 178 patients with PAAD from TCGA database.

Characteristic	Type	*n*	Proportion (%)
Age	≥65	96	53.96
	<65	82	46.07
Gender	Female	80	44.94
	Male	98	55.06
Grade	G1-2	126	70.79
	G3-4	50	28.09
	Unknown	2	1.12
Stage	Stage I-II	168	94.38
	Stage III-IV	7	3.93
	Unknown	3	1.69
T Stage	T1-2	31	17.42
	T3-4	145	81.46
	Unknown	2	1.12
M Stage	M0	80	44.94
	M1	4	2.25
	Unknown	94	52.81
N Stage	N0	49	27.53
	N1	124	69.66
	Unknown	5	2.81

## Data Availability

The data used in this study are all available from the TCGA database (https://portal.gdc.cancer.gov/ (accessed on 4 June 2022)).

## Supplementary Materials

The following supporting information can be downloaded at: https://www.mdpi.com/article/10.3390/ph15111336/s1, Figure S1: The Prognostic Value of Stroma- and Metastasis-Associated Risk Score Model in the Entire Cohort; Table S1: Clinical pathological parameters of 178 patients with PAAD from TCGA database; Table S2: The primers for RT−PCR in this study; Table S3: The primers for RT−PCR in this study; Table S4: Clinical information of RenJi sample.

## References

[1] Connor A.A., Gallinger S. (2022). Pancreatic cancer evolution and heterogeneity: Integrating omics and clinical data. Nat. Rev. Cancer.

[2] Siegel R.L., Miller K.D., Fuchs H.E., Jemal A. (2022). Cancer statistics, 2022. CA Cancer J. Clin..

[3] De Dosso S., Siebenhuner A.R., Winder T., Meisel A., Fritsch R., Astaras C., Szturz P., Borner M. (2021). Treatment landscape of metastatic pancreatic cancer. Cancer Treat. Rev..

[4] Gumberger P., Bjornsson B., Sandstrom P., Bojmar L., Zambirinis C.P. (2022). The Liver Pre-Metastatic Niche in Pancreatic Cancer: A Potential Opportunity for Intervention. Cancers.

[5] Tang D., Wang D., Yuan Z., Xue X., Zhang Y., An Y., Chen J., Tu M., Lu Z., Wei J. (2013). Persistent activation of pancreatic stellate cells creates a microenvironment favorable for the malignant behavior of pancreatic ductal adenocarcinoma. Int. J. Cancer.

[6] Houg D.S., Bijlsma M.F. (2018). The hepatic pre-metastatic niche in pancreatic ductal adenocarcinoma. Mol. Cancer.

[7] Ren B., Cui M., Yang G., Wang H., Feng M., You L., Zhao Y. (2018). Tumor microenvironment participates in metastasis of pancreatic cancer. Mol. Cancer.

[8] Ayala G., Tuxhorn J.A., Wheeler T.M., Frolov A., Scardino P.T., Ohori M., Wheeler M., Spitler J., Rowley D.R. (2003). Reactive stroma as a predictor of biochemical-free recurrence in prostate cancer. Clin. Cancer Res..

[9] Incio J., Liu H., Suboj P., Chin S.M., Chen I.X., Pinter M., Ng M.R., Nia H.T., Grahovac J., Kao S. (2016). Obesity-Induced Inflammation and Desmoplasia Promote Pancreatic Cancer Progression and Resistance to Chemotherapy. Cancer Discov..

[10] Eble J.A., Niland S. (2019). The extracellular matrix in tumor progression and metastasis. Clin. Exp. Metastasis.

[11] Park W., Chawla A., O’Reilly E.M. (2021). Pancreatic Cancer: A Review. JAMA.

[12] Chan T.A., Yarchoan M., Jaffee E., Swanton C., Quezada S.A., Stenzinger A., Peters S. (2019). Development of tumor mutation burden as an immunotherapy biomarker: Utility for the oncology clinic. Ann. Oncol..

[13] Fullar A., Dudas J., Olah L., Hollosi P., Papp Z., Sobel G., Karaszi K., Paku S., Baghy K., Kovalszky I. (2015). Remodeling of extracellular matrix by normal and tumor-associated fibroblasts promotes cervical cancer progression. BMC Cancer.

[14] Kobayashi H., Enomoto A., Woods S.L., Burt A.D., Takahashi M., Worthley D.L. (2019). Cancer-associated fibroblasts in gastrointestinal cancer. Nat. Rev. Gastroenterol. Hepatol..

[15] Desbois M., Wang Y. (2021). Cancer-associated fibroblasts: Key players in shaping the tumor immune microenvironment. Immunol. Rev..

[16] Mao X., Xu J., Wang W., Liang C., Hua J., Liu J., Zhang B., Meng Q., Yu X., Shi S. (2021). Crosstalk between cancer-associated fibroblasts and immune cells in the tumor microenvironment: New findings and future perspectives. Mol. Cancer.

[17] Chen Z., Zhou L., Liu L., Hou Y., Xiong M., Yang Y., Hu J., Chen K. (2020). Single-cell RNA sequencing highlights the role of inflammatory cancer-associated fibroblasts in bladder urothelial carcinoma. Nat. Commun..

[18] Zhang Y., Liu Q., Liao Q. (2020). Long noncoding RNA: A dazzling dancer in tumor immune microenvironment. J. Exp. Clin. Cancer Res..

[19] Schizas D., Charalampakis N., Kole C., Economopoulou P., Koustas E., Gkotsis E., Ziogas D., Psyrri A., Karamouzis M.V. (2020). Immunotherapy for pancreatic cancer: A 2020 update. Cancer Treat. Rev..

[20] Lemaire V., Shemesh C.S., Rotte A. (2021). Pharmacology-based ranking of anti-cancer drugs to guide clinical development of cancer immunotherapy combinations. J. Exp. Clin. Cancer Res..

[21] Notta F., Chan-Seng-Yue M., Lemire M., Li Y., Wilson G.W., Connor A.A., Denroche R.E., Liang S.B., Brown A.M., Kim J.C. (2016). A renewed model of pancreatic cancer evolution based on genomic rearrangement patterns. Nature.

[22] Thomas D., Radhakrishnan P. (2019). Tumor-stromal crosstalk in pancreatic cancer and tissue fibrosis. Mol. Cancer.

[23] Alexander J., Cukierman E. (2016). Stromal dynamic reciprocity in cancer: Intricacies of fibroblastic-ECM interactions. Curr. Opin. Cell Biol..

[24] Nielsen S.R., Quaranta V., Linford A., Emeagi P., Rainer C., Santos A., Ireland L., Sakai T., Sakai K., Kim Y.S. (2016). Macrophage-secreted granulin supports pancreatic cancer metastasis by inducing liver fibrosis. Nat. Cell Biol..

[25] Pedersen S.K., Musher B.L., LaPointe L.C., Tuck M.K., Symonds E.L., Loayza N., Young G.P. (2022). Detection of recurrent colorectal cancer with high specificity using a reporting threshold for circulating tumor DNA methylated in BCAT1 and IKZF1. Cancer.

[26] Basu R., Qian Y., Mathes S., Terry J., Arnett N., Riddell T., Stevens A., Funk K., Bell S., Bokal Z. (2022). Growth hormone receptor antagonism downregulates ATP-binding cassette transporters contributing to improved drug efficacy against melanoma and hepatocarcinoma in vivo. Front. Oncol..

[27] Yin L., Li J., Wang J., Pu T., Wei J., Li Q., Wu B.J. (2021). MAOA promotes prostate cancer cell perineural invasion through SEMA3C/PlexinA2/NRP1-cMET signaling. Oncogene.

[28] Xu X., Zhao Z., Guo S., Li J., Liu S., You Y., Ni B., Wang H., Bie P. (2017). Increased semaphorin 3c expression promotes tumor growth and metastasis in pancreatic ductal adenocarcinoma by activating the ERK1/2 signaling pathway. Cancer Lett..

[29] Subramani R., Lopez-Valdez R., Salcido A., Boopalan T., Arumugam A., Nandy S. (2014). Lakshmanaswamy R: Growth hormone receptor inhibition decreases the growth and metastasis of pancreatic ductal adenocarcinoma. Exp. Mol. Med..

[30] Basu R., Kopchick J.J. (2019). The effects of growth hormone on therapy resistance in cancer. Cancer Drug Resist..

[31] Chen H., Guo Q., Chu Y., Li C., Zhang Y., Liu P., Zhao Z., Wang Y., Luo Y., Zhou Z. (2022). Smart hypoxia-responsive transformable and charge-reversible nanoparticles for the deep penetration and tumor microenvironment modulation of pancreatic cancer. Biomaterials.

[32] Philip M., Schietinger A. (2022). CD8(+) T cell differentiation and dysfunction in cancer. Nat. Rev. Immunol..

[33] Lichterman J.N., Reddy S.M. (2021). Mast Cells: A New Frontier for Cancer Immunotherapy. Cells.

[34] Wasiuk A., Dalton D.K., Schpero W.L., Stan R.V., Conejo-Garcia J.R., Noelle R.J. (2012). Mast cells impair the development of protective anti-tumor immunity. Cancer Immunol. Immunother..

[35] Somasundaram R., Connelly T., Choi R., Choi H., Samarkina A., Li L., Gregorio E., Chen Y., Thakur R., Abdel-Mohsen M. (2021). Tumor-infiltrating mast cells are associated with resistance to anti-PD-1 therapy. Nat. Commun..

[36] Zheng Y., Han Y., Sun Q., Li Z. (2022). Harnessing anti-tumor and tumor-tropism functions of macrophages via nanotechnology for tumor immunotherapy. Exploration.

[37] Cheng H., Huang H., Huang G. (2018). Synthesis and antitumor activity of epothilone B. Eur. J. Med. Chem..

[38] Iliaki S., Beyaert R., Afonina I.S. (2021). Polo-like kinase 1 (PLK1) signaling in cancer and beyond. Biochem. Pharmacol..

[39] Ikezoe T., Yang J., Nishioka C., Takezaki Y., Tasaka T., Togitani K., Koeffler H.P., Yokoyama A. (2009). A novel treatment strategy targeting polo-like kinase 1 in hematological malignancies. Leukemia.

[40] Gohda J., Suzuki K., Liu K., Xie X., Takeuchi H., Inoue J.I., Kawaguchi Y., Ishida T. (2018). BI-2536 and BI-6727, dual Polo-like kinase/bromodomain inhibitors, effectively reactivate latent HIV-1. Sci. Rep..

[41] Ramchandani S., Mohan C.D., Mistry J.R., Su Q., Naz I., Rangappa K.S., Ahn K.S. (2022). The multifaceted antineoplastic role of pyrimethamine against human malignancies. IUBMB Life.

[42] Rubino M.E.G., Carrillo E., Alcala G.R., Dominguez-Martin A., Marchal J.A., Boulaiz H. (2019). Phenformin as an Anticancer Agent: Challenges and Prospects. Int. J. Mol. Sci..

[43] Yu G., Wang L.G., Han Y., He Q.Y. (2012). clusterProfiler: An R package for comparing biological themes among gene clusters. OMICS.

[44] Mayakonda A., Lin D.C., Assenov Y., Plass C., Koeffler H.P. (2018). Maftools: Efficient and comprehensive analysis of somatic variants in cancer. Genome Res..

[45] Yoshihara K., Shahmoradgoli M., Martinez E., Vegesna R., Kim H., Torres-Garcia W., Trevino V., Shen H., Laird P.W., Levine D.A. (2013). Inferring tumour purity and stromal and immune cell admixture from expression data. Nat. Commun..

[46] Hanzelmann S., Castelo R., Guinney J. (2013). GSVA: Gene set variation analysis for microarray and RNA-seq data. BMC Bioinform..

[47] Newman A.M., Liu C.L., Green M.R., Gentles A.J., Feng W., Xu Y., Hoang C.D., Diehn M., Alizadeh A.A. (2015). Robust enumeration of cell subsets from tissue expression profiles. Nat. Methods.

[48] Becht E., Giraldo N.A., Lacroix L., Buttard B., Elarouci N., Petitprez F., Selves J., Laurent-Puig P., Sautes-Fridman C., Fridman W.H. (2016). Estimating the population abundance of tissue-infiltrating immune and stromal cell populations using gene expression. Genome Biol..

[49] Geeleher P., Cox N., Huang R.S. (2014). pRRophetic: An R package for prediction of clinical chemotherapeutic response from tumor gene expression levels. PLoS ONE.

[50] Dobin A., Davis C.A., Schlesinger F., Drenkow J., Zaleski C., Jha S., Batut P., Chaisson M., Gingeras T.R. (2013). STAR: Ultrafast universal RNA-seq aligner. Bioinformatics.

[51] Yang J., Lin P., Yang M., Liu W., Fu X., Liu D., Tao L., Huo Y., Zhang J., Hua R. (2021). Integrated genomic and transcriptomic analysis reveals unique characteristics of hepatic metastases and pro-metastatic role of complement C1q in pancreatic ductal adenocarcinoma. Genome Biol..

