# Supplementary material for: Exploitation and Verification of a Stroma- and Metastasis-Associated Risk Prognostic Signature in Pancreatic Adenocarcinoma
Source: Pharmaceuticals (Basel). 2022 Oct 28;15(11):1336. doi: 10.3390/ph15111336 (PMC9696859; doi:10.3390/ph15111336)
Supplement: Supplementary file 1 [file pharmaceuticals-15-01336-s001.zip › pharmaceuticals-1931139-supplementary.pdf]

pharmaceuticals

Supplementary Materials:

Table S1. Clinical pathological parameters of 178 patients with PAAD from TCGA database.

Covariates	Type	Total	Train	Test	*p*-Value
Age	≤65	94(52.81%)	47(52.81%)	47(52.81%)	1
	>65	84(47.19%)	42(47.19%)	42(47.19%)	
Gender	FEMALE	80(44.94%)	38(42.7%)	42(47.19%)	0.6512
	MALE	98(55.06%)	51(57.3%)	47(52.81%)	
Grade	G1	31(17.42%)	18(20.22%)	13(14.61%)	0.805
	G2	95(53.37%)	46(51.69%)	49(55.06%)	
	G3	48(26.97%)	23(25.84%)	25(28.09%)	
	G4	2(1.12%)	1(1.12%)	1(1.12%)	
	unknow	2(1.12%)	1(1.12%)	1(1.12%)	
Stage	Stage I	21(11.8%)	10(11.24%)	11(12.36%)	0.1806
	Stage II	147(82.58%)	78(87.64%)	69(77.53%)	
	Stage III	3(1.69%)	1(1.12%)	2(2.25%)	
	Stage IV	4(2.25%)	0(0%)	4(4.49%)	
	unknow	3(1.69%)	0(0%)	3(3.37%)	
T	T1	7(3.93%)	4(4.49%)	3(3.37%)	0.8918
	T2	24(13.48%)	13(14.61%)	11(12.36%)	
	T3	142(79.78%)	71(79.78%)	71(79.78%)	
	T4	3(1.69%)	1(1.12%)	2(2.25%)	
	unknow	2(1.12%)	0(0%)	2(2.25%)	
M	M0	80(44.94%)	38(42.7%)	42(47.19%)	0.1777
	M1	4(2.25%)	0(0%)	4(4.49%)	
	unknow	94(52.81%)	51(57.3%)	43(48.31%)	
N	N0	49(27.53%)	25(28.09%)	24(26.97%)	0.9619
	N1	124(69.66%)	61(68.54%)	63(70.79%)	
	unknow	5(2.81%)	3(3.37%)	2(2.25%)	

Table S2. The primers for RT−PCR in this study.

Genes	Primer
18s forward	5′-ATCACCATTATGCAGAATCCACG-3′
18s reverse	5′-GACCTGGCTGTATTTTCCATCC-3′
GHR forward	5′-CCATTGCCCTCAACTGGACTT-3′
GHR reverse	5′-AATATCTGCATTGCGTGGTGC-3′
BCAT1 forward	5′-GTGGAGTGGTCCTCAGAGTTT-3′
BCAT1 reverse	5′-AGCCAGGGTGCAATGACAG-3′
C14orf132 forward	5′-CGCCCAACGAGGACTTCAG-3′
C14orf132 reverse	5′-ATGGCAATCCATAGCAAGACG-3′
SEMA3C forward	5′-TTTGCGTGTTGGTTGGAGTAT-3′
SEMA3C reverse	5′-TCCTGTAGTCTAAAGGATGGTGG-3′
E-cadherin forward	5′-CGAGAGCTACACGTTCACGG-3′
E-cadherin reverse	5′-GGGTGTCGAGGGAAAAATAGG-3′
N-cadherin forward	5′-TCAGGCGTCTGTAGAGGCTT-3′
N-cadherin reverse	5′-ATGCACATCCTTCGATAAGACTG-3′
Vimentin forward	5′-GACGCCATCAACACCGAGTT-3′
Vimentin reverse	5′-CTTTGTCGTTGGTTAGCTGGT-3′

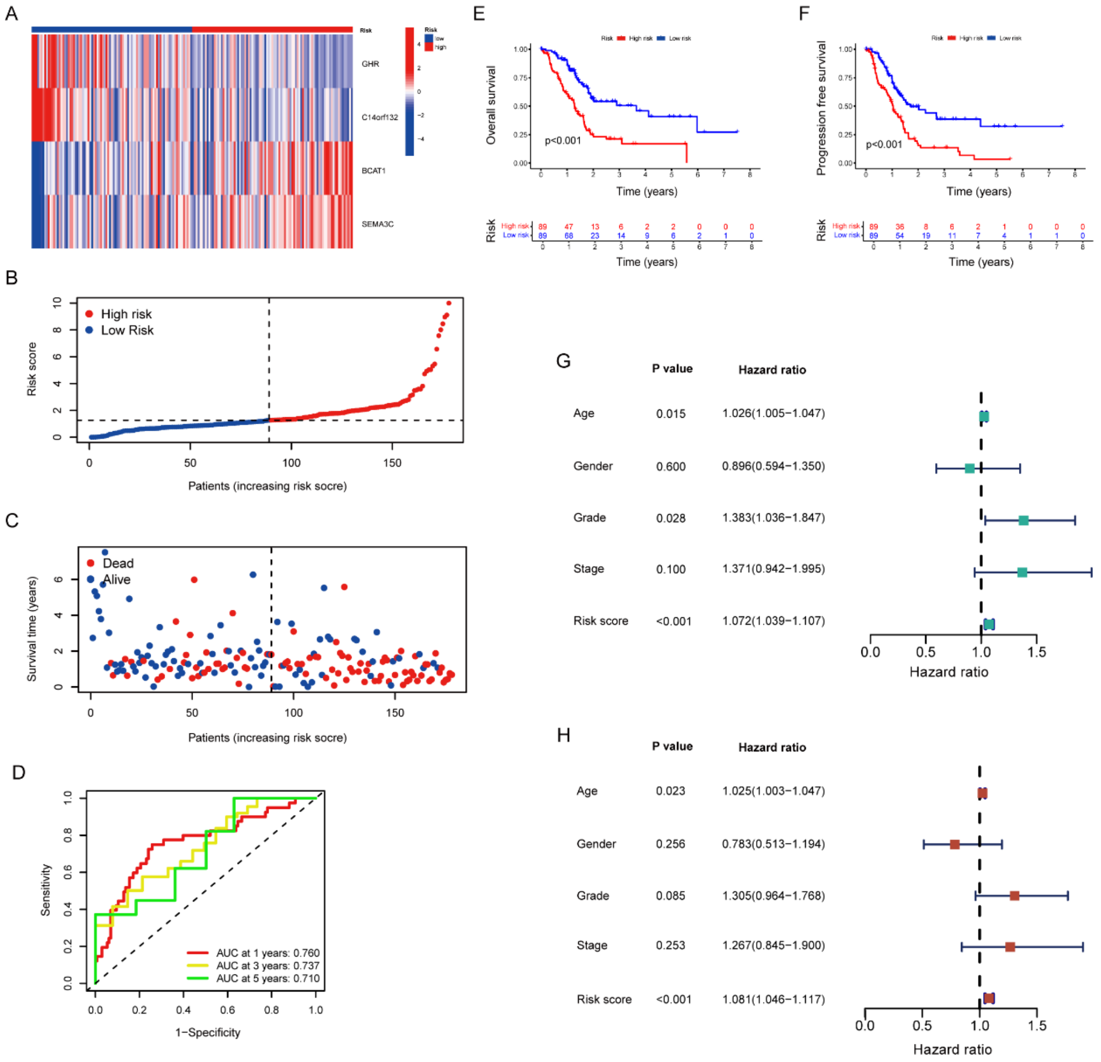

Figure S1. The Prognostic Value of Stroma- and Metastasis-Associated Risk Score Model in the Entire Cohort. (**A**) The heat map illustrated the expression of four genes in each sample. (**B**) The distribution of low- and high-risk samples. (**C**) The correlation of risk score, survival status and

survival time (**D**) ROC curve of the risk score. (**E**,**F**) Kaplan–Meier curve of overall survival and progression-free survival in low- and high-risk groups. (**G**,**H**) The univariate and multivariate Cox regression analysis of clinical characteristics and risk score.

Table S3. The primers for RT−PCR in this study.

Number	Gender	Age	Liver Metastasis	Tumor Size	Metastatic Lesion Diameter	Lymphatic Metastasis	Perineural Invasion	Survival Status	Overall Survival (Month)
1	Male	57	Yes	5.5	2.0	No	No	0	46.0
3	Male	59	Yes	5.5	2.0	No	Yes	1	13.5
7	Female	48	Yes	5.0	1.0	Yes	Yes	1	4.1
8	Male	68	Yes	10.0	2.5	No	No	1	7.3
10	Female	67	Yes	4.0	1.2	No	Yes	1	16.5
12	Male	75	Yes	3.5	0.6	Yes	No	1	17.1
13	Female	53	Yes	2.5	1.4	No	No	0	18.0
14	Male	57	Yes	4.5	1.0	No	No	1	16.1
15	Male	63	Yes	3.0	0.7	No	Yes	1	7.8
17	Female	64	Yes	5.0	1.8	Yes	Yes	1	6.0
18	Male	64	Yes	4.8	1.3	Yes	Yes	1	6.0

Table S4. Clinical information of RenJi sample.

ID	Age	Gender	CEA	CA199	Stage	Risk	Survival Status	Overall Survival (Month)
1	72	FeMale	4.00	10.86	IIA	Low	0	67.4
2	56	Male	6.82	360.20	IV	Low	0	59.8
4	67	Male	1.72	72.01	IIB	Low	0	56.3
5	71	FeMale	17.20	400.30	IV	High	1	15.1
7	62	FeMale	50.92	4110.00	IV	High	1	15.3
8	72	FeMale	3.88	330.90	IV	High	1	5.3
9	69	FeMale	7.59	1.82	IIA	Low	1	50.6
10	65	Male	27.30	303.30	IIB	High	1	46.3
11	45	FeMale	3.51	204.80	III	High	0	61.3
12	69	FeMale	10.87	40.45	III	High	1	19.4
13	63	Male	1.54	132.50	IIB	Low	0	145.8
15	71	FeMale	1.85	161.30	III	Low	1	17.3
16	68	FeMale	2.08	110.70	III	High	1	36.7
17	75	Male	5.09	928.60	IV	Low	1	46.9
19	65	FeMale	2.88	135.70	III	Low	1	138.4
20	71	FeMale	30.83	50.84	III	High	1	37.8
21	68	FeMale	1.21	83.85	III	Low	1	103.9
22	75	Male	2.62	2248.00	III	High	0	51.4
23	53	Male	2.71	216.30	III	Low	1	35.2
24	50	FeMale	2.83	110.60	IB	High	0	31.8
25	68	Male	2.70	98.07	III	Low	0	31.3

26	60	FeMale	2.74	15.06	IIA	Low	1	26.5
27	71	Male	4.51	1.65	IB	Low	1	133.8
29	66	Male	4.55	220.80	III	Low	1	45.0
30	73	Male	1.16	198.00	III	Low	1	43.3
31	65	FeMale	2.82	88.27	III	High	1	37.8
32	76	Male	2.78	10.46	IB	Low	1	25.1
33	79	Male	19.99	82.14	IIB	High	0	22.2
34	63	Male	2.11	695.20	IV	High	1	14.4
35	69	FeMale	1.17	69.87	IIB	Low	0	126.2
36	47	Male	9.80	42.15	IIB	Low	0	125.0
37	64	Male	1.87	1176.00	III	High	1	97.6
39	58	FeMale	2.42	2673.00	IV	High	0	11.2
40	55	Male	27.16	281.40	III	High	1	12.2
41	55	Male	7.59	1526.00	IV	High	0	12.2
42	76	Male	3.50	72.90	IIA	High	0	9.5
43	87	Male	2.33	86.45	III	High	0	9.2
44	62	Male	6.13	328.10	III	Low	0	67.8
45	77	Male	2.13	12.45	IB	Low	1	33.7
